# ‘When it floods, we work on our own’: Exploring factors influencing collective efficacy appraisals for community-level flood measures among urban informal settlements in Suva, Fiji

**DOI:** 10.1002/casp.2808

**Published:** 2024-05-30

**Authors:** Allison P. Salinger, Taylor D'Eramo, Hannah Turner, Autiko Tela, Litea Meo-Sewabu, Maryann G. Delea, Mere Jane Sawailau, Isoa Vakarewa, Sheela S. Sinharoy, on behalf of the RISE Consortium

**Affiliations:** 1 Rollins School of Public Health, Emory University, Atlanta, Georgia, USA; 2 Monash Sustainable Development Institute, Monash University, Melbourne, Australia; 3 School of Public Health and Primary Care, Fiji National University, Suva, Fiji; 4 School of Social Sciences, Western Sydney University, Sydney, Australia; 5 The Carter Center, Atlanta, Georgia, USA; 6 Revitalizing Informal Settlements and their Environments, Suva, Fiji

**Keywords:** climate adaptation, collective action, collective efficacy, flood mitigation, informal settlements, resilience

## Abstract

More than one billion people worldwide are living in urban informal settlements where flood risks are high. Positive collective efficacy beliefs can facilitate community-level adaptive action. This sub-study of the Revitalizing Informal Settlements and their Environments (RISE) trial, aimed to identify social and contextual factors influencing residents' collective efficacy appraisals about their settlement's ability to implement community-level flood prevention, protection and response measures. Forty-two in-depth interviews were conducted in 10 settlements in Suva, Fiji using a photovoice methodology. Thematic analysis was used to elicit key themes, which were then interpreted and contextualized collaboratively with the local field team. The most salient influencing factors were formal leadership, shared needs or benefits, collective identity (whether via shared religion, ethnicity or regional/kinship group), past performance experiences and expectations around collective action. While the data revealed some between-settlement variation on these factors, there was also a large degree of within-settlement variation concerning perceptions of these factors. Community-based flood programming should not be undertaken without first assessing the degree to which participants believe that programme objectives reflect shared needs or will yield shared benefits and whether those objectives warrant collective action according to the community's social expectations for participation and contribution.

## 1 Introduction

### 1.1 Informal settlements and flooding in Fiji

Informal settlements are defined as areas where residents lack security of tenure of the land or dwellings, lack access to basic services and infrastructure, and where hazards exist due to housing structure, geography or environment (UN-Habitat, 2015). More than one billion people worldwide live in informal settlements, and it is estimated that three billion people will be living in these conditions by 2050 (UN-Habitat, 2020). Sustainable Development Goal 11 focuses on making cities and human settlements inclusive, safe, resilient and sustainable (United Nations, 2019). Urban informal settlements are particularly vulnerable to floods due to the hazardous environments in which these settlements are situated as well as general poor construction of houses and land planning, inadequate drainage and high population density (De Risi et al., 2013; Saunders et al., 2016).

Fiji has seen rapid growth of informal settlements in its urban areas (Saunders et al., 2016). Estimates indicate that roughly 15–20% of the population in Suva, Fiji lives in informal settlements (Devi, Lowry, & Weber, 2017); Fiji's Fiscal Review Committee reported in 2023 that 120–140,000 people (15% of all households nationwide) were in informal housing across 170 settlements (Fiji Government, 2023). Many of Fiji's informal settlements are located on the coast and along rivers, leaving them vulnerable to floods and storm surges from frequent cyclones. Fiji is classified as “high” risk on the World Risk Index, which assesses disaster risk based on a country's exposure (i.e., number and proportion of the population exposed) and vulnerability (i.e., susceptibility to disasters and lack of coping or adaptive capacities) (Atwii et al., 2022). The Greater Suva Urban Area, specifically, is exposed to both coastal and riverine flooding (UN-Habitat, 2012).

Rates of sea level rise in the tropical western Pacific, where Fiji is located, are up to four times the global average, which—along with rapid urbanization and coastal development—will increase annual flooding and damage exponentially in the coming decades (Oppenheimer et al., 2019). There is an urgent need for climate adaptation (Oppenheimer et al., 2019), but the pace of upgrading and adaptive action in informal settlements in Suva has been slow (UN-Habitat, 2012). The national government in Fiji is not legally required to provide assistance to informal settlements during disasters (Orcherton, Mitchell, & McEvoy, 2017; Rao et al., 2013). The national government's Climate Change Division recently developed a National Adaptation Plan, which promotes limiting in situ upgrading for “high-risk” informal settlements on the basis that these investments would encourage the growth of such settlements (Fiji Climate Change Portal, 2017).

### 1.2 Collective efficacy and natural disasters

Collective efficacy has been shown to be a key determinant of community resilience to natural hazards (Benight, 2004; Norris, Stevens, Pfefferbaum, Wyche, & Pfefferbaum, 2008) and an enabler of climate adaptation for flooding (Barnett, Graham, Quinn, Adger, & Butler, 2021). Collective efficacy is defined as a group's shared belief in its ability to organize collectively, identify goals or shared interests, develop strategies to address such interests and execute action in pursuit of a common goal or to solve a shared problem (Bandura, 1997a). The concept of collective efficacy comes from social cognitive theory, which states that the behaviours of individuals and groups are influenced by the strength of their efficacy beliefs (Goddard, Hoy, & Hoy, 2004). Positive collective efficacy beliefs yield higher motivational investment in collective action, especially in the face of impediments and setbacks, which in turn increases the likelihood that the group is able to achieve its goal (Bandura, 2000).

Communities and individuals with higher perceived collective efficacy have been found to fare better following natural disasters, including flooding events, and to be better prepared for such events before they occur. Communities with higher collective efficacy have demonstrated more “potential for resilience” against natural disasters and better coping skills post-disaster than those with low collective efficacy (Norris et al., 2008). Studies from high-income countries have shown that individuals with low perceived collective efficacy had higher rates of psychological distress following a flooding event (Benight, 2004) and that a sense of belonging to and confidence in local community enabled climate adaptation to manage flooding (Barnett et al., 2021). However, there are limited studies on collective efficacy in middle-income settings such as Fiji and particularly in urban informal settlements.

Populations most at-risk for adverse consequences from natural disasters are those living in unstable communities with innately low levels of perceived collective efficacy, limited social networks (Weimann & Oni, 2019) and lack of access to the resources those networks might otherwise provide (Trujillo-Pagan, 2007). At the same time, low collective efficacy is associated with less stable jobs and housing as well as a dearth of economic and social resources which could otherwise be leveraged to cope with or adapt to natural disasters (Fay-Ramirez, Antrobus, & Piquero, 2015). It is necessary to develop a better understanding of collective efficacy and related factors that protect against the effects of natural disasters in poor and vulnerable contexts such as urban informal settlements. Furthermore, research shows that overlooking determinants of collective behaviours and social constructs in the implementation of community-based interventions may have negative implications for programme outcomes (Bandura, 2004; Paluck & Shepherd, 2012). Therefore, the purpose of this research was to qualitatively explore the social and contextual factors that influenced residents' collective efficacy appraisals about their settlement's ability to implement flood measures in urban informal settlements in Suva, Fiji.

## 2 Methods

This qualitative study is part of Revitalizing Informal Settlements and their Environments (RISE), a randomized control trial that started in 2017 across 24 informal urban settlements in Makassar, Indonesia and Suva, Fiji. In each city, six settlements were randomized to receive a water-sensitive intervention that involves participatory design and delivery of infrastructure for settlement-scale, decentralized wastewater treatment, water supply, and drainage and flood prevention intended to reduce exposure to faecal contamination (Leder et al., 2021). The remaining six settlements in each city will receive the intervention at a later date. The analysis presented in this paper was conducted using data collected as part of PhD research by HT: in November 2020 a photovoice study was conducted in 10 informal settlements enrolled in RISE to identify and examine flood measures at the individual, household and community levels (Turner, n.d.). Photovoice is a participatory, qualitative research method that uses participant photography and in-depth interviewing to identify key themes (Falconer, 2014).

The study described here was conducted, using the data from HT's flood management photovoice study, with a particular focus on community-level flood measures. In order to comprehensively capture factors influencing residents' collective efficacy appraisals about their settlement's ability to implement community-level flood measures, our methodology (including instrument design, analysis and interpretation) was guided by existing collective efficacy frameworks (Delea et al., 2018; Salinger et al., 2020). These frameworks are each grounded in Bandura's theory of collective efficacy (as described in Section 1.2) (Bandura, 1997a; Bandura, 2000) and validated using quantitative and qualitative empirical data across various country contexts. Specifically, the portion of the interview guide that was focused on community-level measures was designed by AS, SS and MGD using these theoretical foundations and collective efficacy frameworks. The codebook for thematic analysis (described in Section 2.2) included deductive codes reflecting dimensions and sub-constructs of collective efficacy identified in these frameworks that were deemed relevant to the study context and research question.

For the purpose of this study, ‘flood measures’ will be used to refer to prevention, protection and response measures. Prevention measures refer to action taken before a flood to stop effects or to keep a flood from happening, protection refers to measures taken during a flood to keep people and the community safe, and response refers to measures taken after a flood to respond to related effects.

### 2.1 Participant recruitment and data collection

The photovoice study targeted 50 households across the 10 settlements. Households were eligible to participate if they reported having been exposed to flooding directly outside or under the house and/or flood water entering the house in the last 3 months. Within each household, the target respondent was the primary individual responsible for implementing the flood measure(s); however, intergenerational living is common in Fiji and other adult household members frequently contributed information during the interviews. Prior to the interviews, households were given photo-enabled mobile phones and asked to take photographs of ways they prevent, control and mitigate floods, as well as measures they use to protect themselves, their family and their home from flooding. These measures could include individual, household or settlement level flood measures.

Local RISE staff, trained in photovoice methodology, conducted interviews in participants' homes or communal spaces. Interviews were between 30 and 60 min and used the photos that residents had taken as prompts to discuss key themes of interest. Interviews were audio recorded, and interviewers obtained written consent prior to each interview including for interviews to be audio recorded. Consent forms and explanatory statements were provided in English and local languages. Interviews were conducted in iTaukei, Fiji Hindi or English depending on the participants' preference. Recordings were transcribed verbatim and translated to English by RISE team members in Fiji. HT performed quality assurance checks by assessing back-translations from 10% of the interview audio recordings for accuracy and comprehension. The full photovoice methodology is available in Turner (n.d.).

The interview guide included one section that was designed specifically to capture how the community works together to collaboratively prevent, protect or respond to floods and the benefits and challenges of implementing these community flood measures. During this section of the interview, participants were also asked to describe other times their settlement worked together to solve a problem or common issue (i.e., other than for floods) including challenges faced, who initiated and participated in the collective action, and which types of issues were typically solved collectively versus individually.

### 2.2 Data analysis and participatory validation

The study described here uses data from the transcripts only; we did *not* analyse photographs as part of this study. We read each transcript in its entirety, but only analysed data relevant to our research question (i.e., data referencing collective action or collective efficacy). Transcripts were de-identified and uploaded into MAXQDA (VERBI Software, *MAXQDA* 2022) for thematic analysis. Deductive codes were developed based on the interview guide, self-efficacy theory and existing collective efficacy frameworks (Bandura, 1997a; Delea et al., 2018; Salinger et al., 2020). Analytic memos related to key issues and recurring themes were generated on a sample of 14 transcripts across all 10 settlements to inform the development of inductive codes. Once the codebook was developed, with definitions and examples for each code, one analyst (TD) applied all codes to the data set during detailed readings of the transcripts. As new codes were identified, they were iteratively added to the codebook and applied to earlier transcripts. Thematic analysis was used to identify main issues within the data (Hennink, Hutter, & Bailey, 2020). A second analyst (AS) then made within-settlement and between-settlement comparisons of the emergent issues identified in the data (with particular focus on descriptions of collective action, norms, leadership, unity and shared goals) to further establish patterns and better understand the source of variation among resident perspectives and experiences.

In June 2022, one of the analysts (AS) facilitated an in-person workshop in Suva, Fiji with key Fiji-based RISE staff, some of whom were data collectors for the original photovoice study (including MJS and others) and all of whom had extensive experience with community engagement in all settlements enrolled in RISE (including AT, IV and others). The purpose of the workshop was to engage key programme stakeholders in participatory validation (Hennink et al., 2020) of the study findings. Workshop attendees were asked to provide examples of community-level flood prevention, protection and response measures that they had seen or heard about residents implementing in RISE settlements. Attendees were then asked which factors or characteristics of a settlement might influence the degree to which a resident of that settlement feels confident in their community's ability to implement each of the identified measures. After organically eliciting these key factors from the programme staff, we then presented the key issues identified in the data and prompted group discussions around any discrepancies. In December 2022, AS facilitated a second in-person workshop in Melbourne, Australia with a smaller group of the Fiji-based RISE staff (MJS, AT, IV) to present a synthesis of and verify findings from the June workshop. Thus, the workshops allowed us to share research findings with key programme stakeholders, check whether the findings reflected resident experience (as witnessed by programme staff), confirm that the data were saturated, and situate the findings within the context (Hennink et al., 2020). Contributions from the workshops are identified as such throughout the results and discussion sections.

### 2.3 Strengths and limitations

This study obtained data from a mix of genders and a wide age range of participants, which was a strength that allowed for an analysis of different perspectives within study communities. Recruiting participants from 10 communities in Suva, Fiji made it possible to obtain perspectives from multiple informal settlements that have different community-level characteristics such as leadership roles or community groups, which was also a strength of the study. Qualitative data and use of in-depth interviews allowed for analysis of collective efficacy from the emic perspective, which was necessary to develop a rich understanding of the complex social dynamics at play within and between study sites. The participatory, qualitative approach lends the study high internal validity but may limit the external validity or transferability of the findings from our study sample to other populations.

A primary limitation for the study was that the interview guide was originally developed to capture factors that influence residents' confidence in their ability to implement flood-related measures generally and was not specifically looking at beliefs about the settlements' ability to implement *collective* flood measures. Additionally, the interview guide did not provide a definition for the term ‘community’ or ‘settlement’. As there are some discrepancies between socially defined boundaries and the geographic area as defined by RISE, this may have created some uncertainty about exactly which households the interviewer referred to when asking questions about ‘community level flood measures’ or times the ‘settlement worked together’ to protect from floods.

## 3 Results and Discussion

### 3.1 Participant characteristics

A total of 50 participants from 42 households in 10 settlements were interviewed from urban informal settlements in Suva, Fiji. Participants' ages ranged from 22 to 80 years old; 19 participants were female and 13 were male. Participant gender was not specified for 13 households.

### 3.2 Examples of collective flood prevention, protection and response

In Table 1, we outline the types of collective flood prevention, protection and response activities that residents discussed. Many of these activities can be implemented either individually or collectively. For the purposes of this research, we focused on factors influencing residents' confidence in the settlements' ability to implement the measures listed in Table 1 collectively.

### 3.3 Factors influencing flood-related collective efficacy and collective action

#### 3.3.1 Formal leadership: The role of leaders in facilitating flood-related collective action

Formal leadership appeared to facilitate flood-related collective action and promote higher collective efficacy among the participating urban informal settlements in Fiji. Our findings indicate that leadership was most beneficial when residents recognized leaders and imbued them with formal authority.

When the village headman calls out the men and youths move together to try and clean everything out—when something like that [a flood] happens, we will try and take the women and children to a safer place and of course the elders. (Settlement A resident)

This resident highlights the role of the village headman, an example of traditional formal leadership important to the Fijian context. In Fiji, formal leadership, as with many of the concepts discussed below, is inextricably tied to the concept of *Vanua*, which refers to “a people, their chief, their defined territory, their waterways or fishing grounds, their environment, their spirituality, their history, their epistemology and culture”, (Nabobo-Baba, 2006). Social structures with a formal village headman at the top are a key component of an indigenous iTaukei society. However, it is important to note that while some participating settlements were majority iTaukei, others were majority Indo-Fijian and some included at least some residents of both ethnicities.

Reflecting the diversity of the settlements, participants reported different types of formal leadership including village headmen, community committees and chairmen and youth leaders. These leaders performed various roles in facilitating flood-related collective action including (i) organizing collective flood prevention efforts among residents which included scheduling, assigning tasks and so forth, (ii) giving information or instructions for flood protection ahead of a flooding event (e.g., orders for male residents to help families evacuate), (iii) securing or providing resources (e.g., tools and equipment) to implement collective flood measures, (iv) directly implementing flood measures either alongside residents or in instances in which resident participation was low, or checking on households after a flooding event.

Participant (P): We always hold a community clean-up campaign like every quarter in a year. Everybody comes together to clean the drains and compounds and all.

Interviewer (I): So when you come together to clean up who calls for that clean-up campaign? P: The committee in this community. (Settlement B resident)

For this resident, the committee represents one type of formal leadership body with the authority to bring people together for collective action towards flood prevention. Evidence suggests that local community leaders can facilitate collective action in several ways, including enforcing social control (e.g., imposing sanctions when members of the community do not participate in collective action) or linking community members to resources needed to achieve collective goals, among other roles (Salinger et al., 2020). In addition, perceived leader effectiveness and persuasive optimism from leaders can improve members' expectations for the group's ability to work together and thus enhance collective efficacy appraisals (Watson, Chemers, & Preiser, 2001). Research from Pacific Island Countries has also shown that local leadership plays a critical role in prevention, protection and response to environmental hazards, including among disadvantaged urban communities (Ha'apio, Gonzalez, & Wairiu, 2019; McMillen et al., 2014).

Participants in communities without formal leadership noted that a lack of leadership caused disorganization within their settlement, which appeared to create lower collective efficacy appraisals.

What I think is, if there could be a headman of this settlement to go around when there's a heavy rain checking if everyone is ok and if there's a family that needs help—we just need someone to oversee. (Settlement C resident)

This resident preferred to have a formal leader to oversee the community and provide assistance in times of need, including during flooding events. However, there were also some communities that had formal leaders who received limited support from residents. In these communities, residents tended not to follow instructions or heed warnings from leaders. In settlements with ‘traditional’ or ‘village-like’ hierarchical social structures, formal leaders tended to be recognized and imbued with authority by the collective. These were often majority indigenous Fijian or iTaukei communities in which residents are either indigenous to the land on which they live or have relocated there as a collective, bringing with them their existing social structures. In some other informal settlements (that were more heterogenous or that did not have ‘traditional’ social structures) formal leaders were not perceived as legitimate leaders by all residents.

I then became the committee leader but the support for me only lasted for about a month. […] All I know is that I was chosen to lead this committee. I asked them what's going on, but there's usually no response. (Settlement D resident)

This speaker does not receive support from constituents despite holding a formal leadership role (committee leader). This phenomenon may be related to a continued deterioration of traditional leadership structures in urban Fiji. Specifically, urbanization has led to the reduction in importance of traditional (or chiefly) authority and the erosion of customary governance mechanisms for citizens to engage with leaders (Geaves & Penning-Rowsell, 2016; Keen et al., 2022; Williksen-Bakker, 1995). For some informal settlements from our study, this may have created a leadership vacuum whereby the lack of formal leadership promoted a sense of disorganization. In other settlements, this may lead to a parallel system of government administration and chiefly leadership, creating confusion about legitimate authority, which limits the community's ability to respond to and prepare for disasters such as floods (Keen et al., 2022).

#### 3.3.2 Shared needs or benefits: Perceived environmental risk and flooding measures as ‘common pool resources’

In many settlements, residents reported that sections of the community that were affected by tidal flooding felt confident about their ability to work together to implement collective flood measures. These residents were motivated by shared needs to work collectively to prevent future flooding and help each other clean up after floods. However, other residents from the same settlements (usually those living on higher ground) would not participate because they did not face the same challenges.

I: Was there a time when the households here at [Settlement] would work together as a community, like […] preventative measures in avoiding flood as a community?

P: No. For me, I would say it's just the households here at the bottom because those that are up on the top do not go through these challenges. (Settlement E resident)

This resident describes fragmented implementation of flood measures in their settlement. This was common to many study settlements and may be problematic as diverting water from one area of the settlement can have negative implications for other areas. Engineering works intended for flood prevention can be considered public goods insomuch as anyone living in the protected area can benefit (Geaves & Penning-Rowsell, 2016). However, due to the spatial aspect of flooding, those who live within the boundaries of the “benefit area” but who did not seek to benefit from the flood measure have “little incentive to engage” (Geaves & Penning-Rowsell, 2016). For these reasons, some have argued that flood management can be better understood as a common pool resource, meaning that flooding mitigation is a shared resource that is ‘rival’ because floodwater can be channelled from one area to another and ‘subtractable’ because freeriding or benefitting from flood management without contributing to it leaves more water for others to deal with (Cowen & Delmotte, 2020; Ostrom, 1990; Ostrom, 2010). Our results support this framing of flood management as a common pool resource.

Now we've been requesting for the soil truck to deploy another soil deployment here so we can continue with the land fill. All of us that stay at the back we've been trying to fill ours [area outside their house] because if we fill it up, then the water will flow over to that side [the other side of the community]. Hence the reason we want to fill all the land area at the back. We have to attempt doing this because those in the front [pointing towards the houses that are in front, facing the main road] they are hardly affected by the waves. Only when there is heavy rain and tidal waves then it can affect those in front. (Settlement F resident)

This speaker provides an illustrative example of how differences in needs (based on location within the settlement and resultant susceptibility to flooding) disincentivizes whole-of-community collective action. Residents from a few settlements also differentiated between collective efficacy appraisals for working together during cyclones versus preventing or responding to tidal flooding. While tidal flooding only affected certain areas of the settlement, cyclones had the potential to affect the whole community. Residents reported that it was common for the entire community to work together or have responsibility for dealing with whole-of-community needs during cyclones even in communities that had fragmented implementation for dealing with tidal flooding.

When it rains heavily due to a cyclone coming, the village would be under water and so it would be the responsibility of the whole village rather than if it was just a high tide and a house would go under water then the head of the house will do his responsibility in cleaning up his house and we would just keep a look out for our own houses. (Settlement A resident)

The distinction drawn by the speaker between cyclones and high tides demonstrates that collective action and collective efficacy appraisals may be highly specific depending on the type of flooding event or one's residence in a certain area within the community. Other research from informal settlements in Fiji suggests that perceived risk of a variety of environmental hazards may differ greatly even between otherwise similar settlements (Devi et al., 2017). Our research takes this one step farther by demonstrating that even residents of the *same* settlement may not perceive shared needs or benefits for flood prevention, protection or response.

#### 3.3.3 Collective identity: Ethnic polarity in Fiji and links to religion, kinship and land tenure

Differences in ethnic, religious or kinship groups within study settlements were often seen as barriers to collective action and limited residents' perceived collective efficacy. Specifically, interviewees felt that residents from different ethnic backgrounds (termed ‘races’ locally) had different customs around collective action and social support, which had implications for residents' beliefs about their ability to work across ethnic groups.

There are some people that will have their own ways while there are some who are so committed to the works of the community. It exists because people of different races live here. Sometimes when a meeting is held, when the meeting is called only a few are present. (Settlement F resident)

This resident perceives that heterogeneity in the community across ‘races’ poses a challenge for collective action; this finding is not entirely surprising given the historical divisions that exist among these groups in Fiji. Indigenous Fijians or iTaukei are nearly all Christians while 80% of Indo-Fijians are Hindu and because intermarriage is uncommon, kinship groups also fall along ethnic divides (Norton, 2000). During colonization, the British brought Indians to work on sugar plantations in Fiji as indentured labourers while simultaneously codifying the political power of indigenous Fijians (McCarthy, 2011; Meller, 1984). Since independence in 1970, the country has seen four coups (the most recent occurring in 2006) driven in large part by the ethnic divide between iTaukei and Indo-Fijians (McCarthy, 2011) and even during more stable periods, “reciprocal denigrative stereotyping persists” (Norton, 2000).

Residents who were members of the community church reported benefitting from church-led efforts while others were excluded, although these were not necessarily flood-related efforts. One resident described how even differences in Christian denomination could act as a barrier to successful collective action:

There are many church denominations that live here, but we do not work together. It's us the Methodist denomination that is more dominant here. We know that it is us who carry out the responsibilities in [Settlement]. I have never seen a time where everyone came together to solve a problem. (Settlement C resident)

In study settlements where the majority of residents viewed each other as ‘related’ or ‘family’ (whether because of actual blood or marriage relations or because of a shared ancestral connection to an area or island of origin), these kinship social ties facilitated collective action.

Because in this place we are all related, we usually help each other in relocating to higher places. There are a lot of people who experience flooding here; they look for houses with longer posts to evacuate to. Houses that are down there are relocated to houses with longer posts for shelter. (Settlement G resident)

This speaker describes an example of collective flood protection (evacuation), which they perceive to be facilitated or motivated by the kinship ties within the settlement. In Fiji, land rights can be obtained via *Vakavanua* (i.e., informal land agreements whereby settlers are granted access to native land held communally under ‘customary’ ownership by an indigenous clan), which ties land rights to kinship group membership and therefore to ethnicity and religion (Ben & Gounder, 2019; Kiddle, 2010). This practice results in iTaukei landowners having greater access to land via ‘traditional approaches’ than their non-iTaukei counterparts (Ben & Gounder, 2019) and informal settlement residents necessarily having existing connections prior to moving into a settlement (Kiddle, 2010). These social connections can facilitate collective action to protect against natural disasters such as floods (Amoako, 2018; Aßheuer, Thiele-Eich, & Braun, 2013; Yila, Weber, & Neef, 2014). Evidence from Fiji shows that individuals or communities with larger social networks were in a better position to coordinate recovery and response efforts when faced with flooding disasters (Yila et al., 2014).

Households that did not have kinship ties or shared ancestral connection to an area/island of origin in Fiji had less participation in collective action and lower collective efficacy appraisals. One resident articulates the challenges for their family because they lack kinship ties in a community where most residents are “relatives”:

Some of the people they're relatives and that's how they help one another, but for us from other areas is very difficult. (Settlement E resident)

In some cases, these differences simply created within-settlement variation in collective efficacy perceptions and experiences of collective action whereby members of the in-group(s) had higher collective efficacy appraisals and more positive experiences of collective action. In other cases, the differences in ethnic, religious or regional/kinship groups were seen as barriers to collective action. Strong intragroup networks in the absence of bridging (i.e., intergroup) networks can act as a barrier to social cohesion in heterogenous communities (Klein, 2016). This may help to explain why we saw evidence that ethnic and religious differences were barriers to flood-related collective action in the absence of strong intergroup ties. Thus, issues of ethnicity, religion, kinship and land tenure are inextricably linked in the Fijian context making the establishment of a collective identity that transcends Fiji's deeply ingrained, ethnically-informed in-groups a major challenge for inclusive collective action among heterogenous communities.

#### 3.3.4 Past performance experience: Experience with collective action influences collective efficacy appraisals

Past experiences with collective efforts for flood measures appeared to influence future collective action and collective efficacy appraisals. Participants who reported positive mastery experiences with working together for flood measures discussed how working together had a unifying effect, strengthening the community's ability to work together in the future. Participants who reported negative past performance experiences reported low collective efficacy appraisals and appeared unmotivated to try to work towards collective flood measures.

Many residents described high outcome expectations (Bandura, 1997b) related to implementing community level flood measures, meaning residents tended to believe that if they were able to work together for flood related measures, they could achieve better results and effectively protect the community from floods, improve their surroundings and community wellbeing, and benefit future generations. When asked about benefits of working together to implement flood measures, residents said they would accomplish tasks more quickly, cover more ground, learn skills from others and ease or reduce the overall workload.

However, in some communities, even when residents had high outcome expectations, they described low efficacy expectations: although they believed collective flood measures could be effective, they had low confidence in their settlement's ability to organize and/or mobilize residents towards these goals. These residents tended to either describe failed past attempts at collective action or a lack of community experience engaging in collective action around flooding. Thus, negative performance experiences lowered collective efficacy appraisals and were a barrier to collective action for flood measures. When participants tried to engage in collective action with other community members and failed or had a negative experience, these residents sometimes stopped trying to get others to participate and often turned to more individual or household-level flooding solutions.

I: Can you tell me if you have done any flood response measures as a community?

P: The use of the tires—we did it together as a community but we did not finish it because everyone took it lightly. The unity of the community and working together of the family was not there and so we will only be able to avoid flood by making the tires better […] Unity of working together is hard and people rather just do their own things. (Settlement H resident)

This resident provides an example of a negative past performance experience in which a collective flood protection measure went unfinished due to a lack of “unity of the community”. Alternatively, positive past performance experiences (i.e., instances in which communities were able to come together and successfully accomplish a goal) appeared to promote more positive efficacy expectations and further success in implementing collective flood measures.

I: Did you happen to have worked together as a community?

P: Yeah […] We would clean up. We would pick up the rubbish from the places here. When there was a flood or cyclone, we would go to the individual houses to see what was bad. For those staying at the bottom to move up. […] It brought together unity in the community, the togetherness and the working together that unites people together. (Settlement I resident)

This resident explains how working together and overcoming challenges had a unifying effect and strengthened the community's ability to work together in the future. These findings are consistent with Bandura's framework for efficacy expectations, which includes positive mastery experiences as a key dimension (Bandura, 1997b). Similarly, in urban informal settlements in Ghana, residents were able to learn from past experiences and respond to flooding when needed based on their prior knowledge (Amoako, 2018).

#### 3.3.5 Expectations around collective action: What tasks warrant collective action and who should participate

The degree to which collective action was expected varied between settlements. While residents of some settlements explained that working together was core to their community, others described the expectation that family and household needs were prioritized above community needs.

This community is well known for working together. If there's a problem or a funeral in one of the families, we're all ready to assist. A contribution of $2 from each family, and this is all because it's known that people work together and care for each other's needs. (Settlement F resident)

For this resident, the expectation for collective action was part of the community identity. Similarly, community norms or socially embedded rules concerning which behaviours are or are not acceptable among members of the collective have been identified as either dimensions of collective efficacy or contributors to collective behaviour in other contexts (Ahern, Galea, Hubbard, & Syme, 2009; McGranahan & Mitlin, 2016; Salinger et al., 2020). Throughout this section, we explore sources of variation in social expectations for flood-related collective action in the study settlements.

In many communities, residents had specific expectations for who should be involved in collective implementation of flood measures and/or who was ‘allowed’ to not participate in collective action generally.

For the community members, during natural disasters like this we work together. […] Especially for the elders who cannot do anything […] and the mothers will try and stay with their children and the elders. We will do the work, we as fathers and the young men. (Settlement A resident)

As exemplified by this quote, residents often described collective flood measures being implemented specifically by men and boys. There were also a couple of communities in which residents expected community committees to implement community-level flood measures directly (see the Formal Leadership section). The data highlights the critical role that youth (i.e., adolescents) played in flood-related collective action in many settlements. One resident described generally low expectations for implementation of collective flood measures in their community except for instances of youth-led activities:

I: When you work together to solve a problem or common issue, is every household and resident involved in the effort or only some people or households participate?

P: Yes, when we work together during cyclones, the youths usually come outside and the family stays indoors. (Settlement I resident)

There also seemed to be a social expectation in nearly all communities that there would always be at least some households that refused to participate in collective action generally. Reasons that residents gave for this lack of participation included inability of certain households to contribute money or other resources, time constraints (households that were “closed” during the day because all members were at school or work) and household structure or gender make-up (i.e., households with no or few men were less able to contribute to collective efforts).

Yes for those who can help if you have it you give it, if not then there's nothing to do. If they say $10 and all they have is $7 then that's what they’ll give, everyone always assists in whatever ways they can. (Settlement G resident)

As is clear from this quote, lack of participation for the above-mentioned reasons (including inability to contribute money as mentioned by this resident) appeared to be ‘allowed’ and did not necessarily act as a barrier to collective action for those households who were able to participate. Interviewees also explained that it was common for a few households to simply refuse participation, even if they were otherwise capable of participating.

I think not only in our community but other communities there are some who listen and others who don't [laughter]. It's just the mentality of people. Some are like that—do not want to be taught, do not accept new teachings that come in. So it's always a barrier. (Settlement H resident)

This resident perceives that these refusals to participate are attributable to residents' “mentality”, but appears to normalize this phenomenon which they believe is common not only in their community, but in others as well. The RISE research team in Fiji told us that these households may be visited by the settlement leader to encourage participation and/or presumed to be refusals in the future and therefore not invited to participate. However, residents did not report strong social sanctions and instead said that they felt like they could not ‘force’ others. According to the social norms literature, this lack of social reaction to a collective behaviour indicates relatively weak normative beliefs (Bicchieri, 2017).

##### The role of perceived tenure security and sense of belonging

Settlements with more secure land tenure tended to have a stronger sense of belonging which in turn motivated and created expectations for residents to participate in collective action. According to the team in Fiji, settlements with indigenous ties to the land also tended to follow indigenous (‘traditional’ or ‘village-like’) social structures defined by kinship ties and strong formal leadership, both of which were said to play a role in enforcing expectations that community members contribute to collective action. This social structure and indigenous worldview also dictates expectations and obligations for contributions to the collective.

I: When you work together to solve a problem, is every household and resident involved in the effort?

P1: I don't think so

P2: Because this place is not like a village. If it was a village, people would cooperate. (Settlement C resident)

This resident speaks to the strength of social expectations and obligations that are in place in ‘village-like’ communities. In the absence of indigenous social structure and sense of place/belonging, this resident was not confident that their community would be able to work together towards shared goals. Links between land tenure and collective action are clearly corroborated by existing literature. Specifically, evidence from Fiji's informal settlements suggests that perceived tenure security creates incentive to invest in community development, including implementing adaptations to cope with natural hazards and climate change (Orcherton et al., 2017). Research has found that, across both state held land and native land settlements (i.e., those acquired via *vakavanua*), Indo-Fijians had more negative perceived tenure security than iTaukei participants (Kiddle, 2010). This may create less motivation to participate in collective action among Indo-Fijian residents.

The complexity of land tenure in urban informal settlements in Fiji goes far beyond the ‘state’ and ‘native land’ dichotomy. The informal nature of land acquisition in Fiji has led perceived tenure security to depend heavily on informal relationships between tenants and landowners, especially in native land settlements (Ben & Gounder, 2019; Kiddle, 2010). In some RISE settlements, the church purchased communal land-use rights and is then involved in decision-making around which tenants are allowed to occupy the church-owned land. In other settlements, plots (although owned communally by clan landowners) have been purchased by individual families, which has disincentivized settlement-scale collaboration for flooding and, according to RISE staff, led to household-level flood mitigation measures that redirect additional water to neighbouring plots.

##### Domain-specific collective efficacy and obstacles to flood prevention measures

Residents also varied in terms of the types of tasks or goals for which they expected their community to work together. Many participants expressed that it was normal for members of their settlement to come together for weddings, funerals, and building roads and houses. However, as one resident explained, these expectations did not always translate to expectations for working together around flood measures:

I: Do the residents work together to implement these flood response measures that the community has implemented together at a community level?

P: Yes, we work together

I: During a flood? Or you work together on other things?

P: Yes, other things. When it floods, we work on our own. (Settlement I resident)

Further, residents from a few communities reported that while they tend to work at the family/household level on flood *prevention* measures, they did have expectations that the community would come together to help with *protection* and particularly evacuation. Other scholars have posited that in terms of adaptive action to protect from natural disasters, residents of informal settlements in Fiji are more likely to be focused on immediate concerns rather than long-term timeframes (Orcherton et al., 2017). State agencies in Fiji have no obligation and little incentive to intervene in informal settlements (Kiddle, 2010) and this lack of access to external resources or support underscores the challenges faced by residents in undertaking larger scale preventative flood measures.

I: There was no [flood] response measure implemented together at community level?

P: No, we do our own […] the only time we worked together was regarding the boat, when they came to assist in taking us across with our belongings and family. (Settlement E resident)

For this resident, social expectations for collective action apply to flood protection (evacuation), but do not apply to flood response. This type of domain-specific collective action may be related to domain-specific collective efficacy appraisals. Domain-specific collective efficacy has been observed in other studies (Salinger et al., 2020) and is a well-established concept in the self-efficacy literature, which argues that one's confidence in their ability to achieve a goal or task in one domain may or may not be related to confidence in other domains (Chen, Gully, & Eden, 2016; Donald, Koolwal, Annan, Falb, & Goldstein, 2020; Eden, 1988).

Overall, this variation in social expectations for flood-related collective action is an important consideration for programme implementers as they design community-level interventions, as it demonstrates that it is not enough to simply identify ‘cohesive’ communities and expect that community members will work together to make progress towards collective goals defined by the programme. Instead, it may be necessary to first establish the specific goal or task as something that warrants collective action and for which residents feel others in the community would expect them to participate or contribute.

## 4 Conclusion

This study explored the social and contextual factors that influenced residents' collective efficacy appraisals about their settlement's ability to implement flood measures in urban informal settlements in Suva, Fiji. The most salient influencing factors in these settlements were formal leadership, shared needs or benefits, collective identity (whether via shared religion, ethnicity or regional/kinship group), past performance experiences, and expectations around collective action, particularly around which tasks or goals warrant collective action and who is expected to participate. The data revealed both between-settlement and within-settlement variation concerning the factors that influence collective efficacy appraisals and collective action. Residents within the same settlement often had conflicting collective efficacy appraisals and descriptions of experiences with collective action. Other research in informal settlements in Fiji has noted that settlements vary substantially in their “sociocultural dimensions” (Kiddle, 2010; Orcherton et al., 2017) and recommends better understanding of the “social characteristics” of settlements in order to inform urban planning to deal with environmental threats (Devi et al., 2017). Our research demonstrates that these complex social dimensions may contribute to differences in lived experiences and beliefs between and within settlements and, hence, to the potential for community-level flood measures to be successful.

### 4.1 Implications and recommendations

Collective efficacy has been shown to be a key determinant of community resilience to natural hazards (Norris et al., 2008). It is, therefore, vital to understand the mechanisms underlying collective efficacy appraisals, especially in informal settlements where residents are most at risk for damage due to natural disasters like floods (UN-Habitat, 2012) and where reliance on community-based solutions may be more pronounced due to limited access to government services, infrastructure and support (Kiddle, 2010). Community-based development programmes that have failed to account for the social dynamics and behavioural determinants inherent in their theories of change have shown poor outcomes (Bandura, 2004; Paluck & Shepherd, 2012)—a reality that programme participants living with the threat of sea level rise and increased flooding damages can hardly afford.

Against this urgent backdrop, researchers and programme implementers should consider the key barriers and enablers identified in this study prior to the design or implementation of community-based flood prevention, protection and response research or programming. Formative research to inform programme design and/or contextualize future research is necessary to understand the extent to which the factors identified in this study enable or hinder collective action in other contexts as well as to identify any additional or alternative factors that may be relevant in different social contexts. Formative research should be intentionally designed to capture differences between *and* within settlements and should assess the degree to which participants believe that programme objectives reflect shared needs or will yield shared benefits and whether those objectives warrant collective action according to the community's social expectations for participation and contribution. Where this is not the case and/or where collective efficacy appraisals are weak, programme implementers may consider selecting alternative intervention techniques that do not require collective action, incorporating intervention techniques that aim to strengthen collective efficacy appraisals (Delea et al., 2018), or linking programme objectives with other public goods for which communities are willing to collaborate (Reese et al., 2017). Future research should further differentiate between factors informing collective efficacy appraisals for different types of flood measures. This is particularly necessary for flood-related programming as smaller scale or household-level measures may worsen flooding conditions or damages due to flooding for others in the area.

Policymakers should provide resources for and enforce the delivery of substantive in situ upgrading of informal settlements in flood-prone areas. It is not enough to provide “basic lifeline services” for these communities as this reinforces poor security of tenure and, therefore, provides no incentive for communities to utilize higher quality housing stock or implement expensive adaptive measures. Policies must account not only for physical and legal changes associated with upgrading settlements, but also account for the role of perceived tenure security, which has been shown elsewhere and in our study to have important implications for willingness or ability to implement collaborative flood measures in these contexts (Fiji Climate Change Portal, 2017; Kiddle, 2010; Orcherton et al., 2017; Rao et al., 2013).

## Figures and Tables

**Table 1:** Collective flood prevention, protection and response measures identified in interview transcripts.

Flood measure	Examples identified in the data
Collective Prevention^a^	Digging or deepening drainage ditches Cleaning rubbish around the community and especially in drainage ditches Clearing the areas around culverts Weeding tall grasses (to allow water to flow over the area more easily) Planting mangroves Filling in depressions with sand Constructing barriers (e.g., stacking bags of sand and coconut husks, piling mud and dirt removed from drainage ditches and in some cases planting vegetation in the mud and dirt, and placing corrugated metal as flood barriers) Some interviewees described “talks” or “plans” for the construction of a floodwall, but it was unclear from the data whether these plans were executed and to what extent
Collective protection	Evacuation—Men and boys assisted women, children and elderly with getting to evacuation centres Collaborating to help neighbours pack up before a flood, secure their belongings or put up shutters (also mostly men and boys) Placing old tires around the settlement to act as an elevated footpath when the area became inundated Stilting of houses was sometimes described as a collective effort in which neighbours provided assistance
Collective response	Working together to clean up debris and rubbish after a flood (one settlement specified that their clean-up campaign was aimed to destroy breeding places for mosquitoes following floods in their community) Helping neighbours rebuild houses that had been damaged or destroyed Working together to remove a blockage in the drainage system to allow floodwaters to recede

a While prevention measures appear to have the greatest variety in terms of examples, they were not necessarily the most commonly implemented type of flood measure.

## Data Availability

The data that support the findings of this study are available from the corresponding author upon reasonable request.
